# Editorial: Immune dysfunction in nephrotic syndrome - recent advances and new roads ahead

**DOI:** 10.3389/fimmu.2022.985925

**Published:** 2022-08-02

**Authors:** Barbara Seitz-Polski, Vincent Audard, Gian Marco Ghiggeri, Nicola M. Tomas

**Affiliations:** ^1^ Centre de Référence Maladies Rares Syndrome Néphrotique Idiopathique, CHU de Nice, Université Côte d’Azur, Nice, France; ^2^ Unité de Recherche Clinique de la Côte d’Azur (UR2CA), Université Côte d’Azur, Nice, France; ^3^ Laboratoire d’Immunologie, CHU de Nice, Université Côte d’Azur, Nice, France; ^4^ Service de Néphrologie-Dialyse-Transplantation, CHU de Nice, Université Côte d’Azur, Nice, France; ^5^ Assistance Publique des Hôpitaux de Paris, Hôpitaux Universitaires Henri-Mondor, Service de Néphrologie et Transplantation, Centre de Référence Maladie Rare “Syndrome Néphrotique Idiopathique”, Fédération Hospitalo-Universitaire, Innovative Therapy for Immune Disorders, Créteil, France; ^6^ Univ Paris Est Créteil, Institut National de la Santé et de la Recherche Médicale (INSERM) U955, Institut Mondor de Recherche Biomédicale (IMRB), Créteil, France; ^7^ Laboratory on Molecular Medicine, Istituti di Ricovero e Cura a Carattere Scientifico (IRCCS) Istituto Giannina Gaslini, Genoa, Italy; ^8^ Division of Nephrology, Dialysis, Transplantation, Istituti di Ricovero e Cura a Carattere Scientifico (IRCCS) Istituto Giannina Gaslini, Genoa, Italy; ^9^ III. Department of Medicine, University Medical Center Hamburg-Eppendorf, Hamburg, Germany

**Keywords:** nephrotic syndrome, membranous nephropathy, minimal change disease, focal-segmental glomerulosclerosis, autoantibodies, rituximab

## Introduction

**Figure 1:**
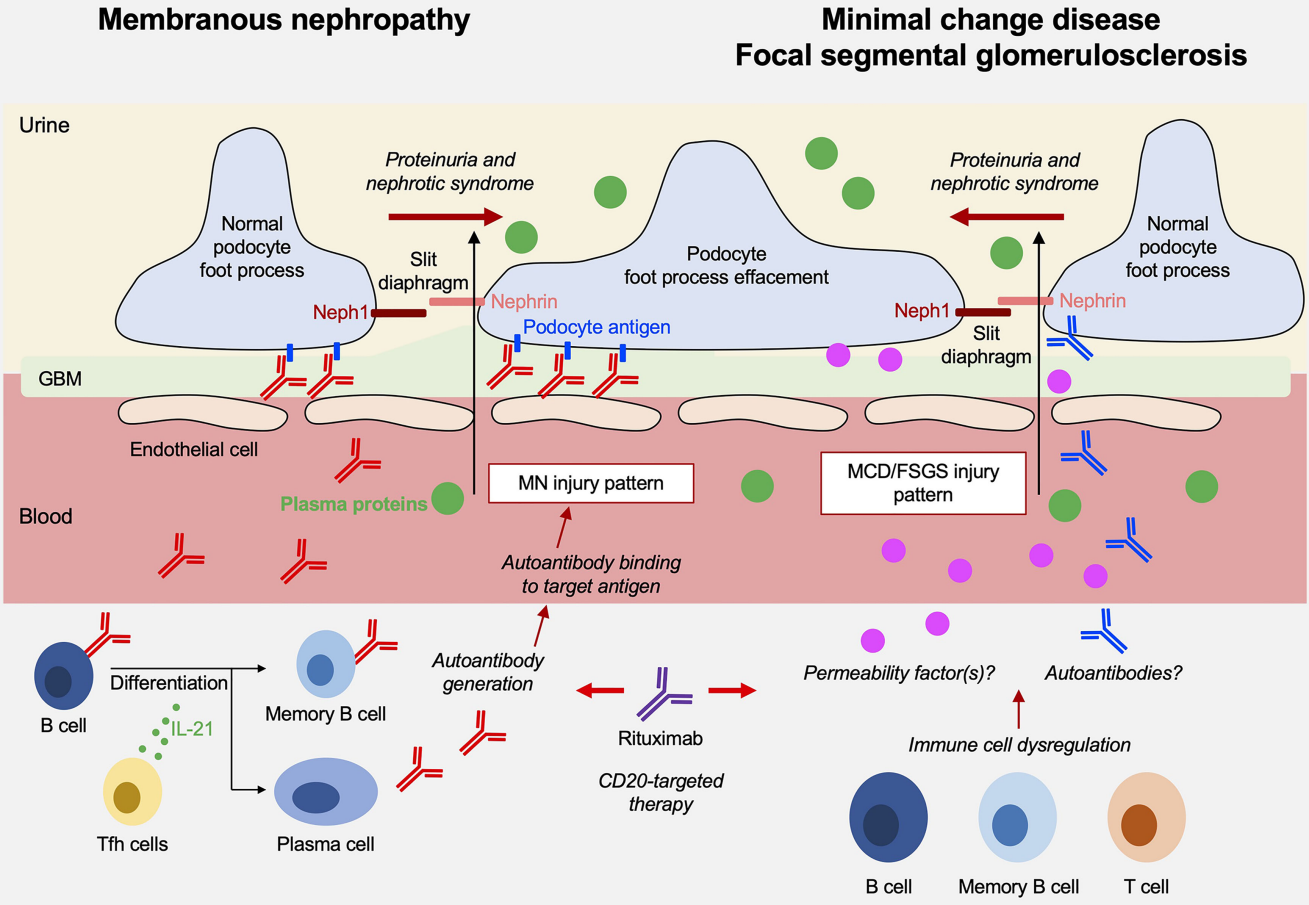
Overview on the pathogenesis of membranous nephropathy (MN) and minimal change disease (MCD)/focal segmental glomeruloscleroses (FSGS).

Nephrotic syndrome (NS) is a clinical condition characterized by proteinuria, a reduction in serum levels of albumin and other proteins, edema, hypercholesterolemia, and often predisposition to thrombosis. Prerenal acute kidney injury or acute tubular necrosis may occur in most serious cases. NS occurs in children and adults as a consequence of specific pathological conditions that have age-specificity and that are characterized by certain common histological findings, such as the effacement of podocyte foot processes and loss of the slit diaphragm architecture, leading to a disruption of the glomerular filtration barrier and loss of plasma proteins into the urine (**Figure 1**). The resulting clinical signs and symptoms are consequences of the urinary loss of transport proteins, coagulation factors, metabolites and others.

Underlying pathological renal lesions associated with NS have been categorized over the past decades. Membranous nephropathy (MN), minimal change disease (MCD) and focal segmental glomerulosclerosis (FSGS) represent the most common primary glomerular disorders underlying a nephrotic syndrome and now build the basis for a morphologic classification that has the merit to create a common reference for clinicians and a starting point for studies on therapies. The underlying molecular mechanisms are various and include genetic modifications, degenerative and infectious causes, inflammatory conditions, and autoimmunity, but unknown causes represent an empty chapter that still needs be filled despite almost 50 years of research in the field. It is remarkable that diseases characterized by different pathology backgrounds (e.g. MN, MCD, FSGS patterns) are still often treated with the same drugs. This highlights the need to define NS and its major causative entities on a deeper molecular level and justifies a special issue in *Frontiers in Immunology* dedicated to ‘Immune dysfunction in nephrotic syndrome’.

The indications of the special issue were wide but have been intended by Authors to be limited to those conditions with a clear immunologic origin and MN had the major interest. This is justified by recent discoveries of disease-specific circulating autoantibodies (1–10). MCD/FSGS and more in general childhood idiopathic NS were second in the number of papers. The pathogenesis of MCD and FSGS have not yet been resolved, and the major focus was on immunomodulatory therapies targeting CD20, which have been developed over the last few years which and are linked with the production of antibodies. They suggest a key role of B lymphocytes in MCD/FSGS pathogenesis that deeply has modified our knowledge about these two glomerular diseases. Points of interest that bridge MN with MCD/FSGS are the recent discovery of anti-nephrin antibodies in the latter condition (11) that would become, if the finding will be confirmed, an antibody-mediated autoimmune disease, further justifying the use of anti-CD20 monoclonal antibodies.

## Membranous nephropathy

Primary MN is one of the most frequent causes of nephrotic syndrome in adults without diabetes. It is histologically characterized by granular deposition of IgG and complement factors at the glomerular filtration barrier, a finding that has long indicated a critical role of autoantibodies in the pathogenesis of this disease. In 2009, the identification of antibodies targeting PLA2R1 – which represents the main antigenic target in MN – confirmed this pathophysiological concept (2). Since then, a number other renal autoantigens were identified using different methodological approaches. These new autoantigens are frequently associated with unusual clinical presentations as well as secondary etiologies (lupus, autoimmune diseases, neoplasia…) that require special attention and therapeutic measures. These discoveries were reviewed by Caza et al. in this Research Topic. In addition to PLA2R1 (60-70% of all MN patients) (2), the list includes other podocyte antigens such as THSD7A (2-4% of patients) (3), HTRA1 (6) and SEMA3B (12), as well as NELL1 and PCDH7 (5), which are not expressed by normal podocyes. Finally, a few new antigens are associated with other clinical conditions, such as EXT1/2 (13), NCAM1 (10) and TGFBR3 (9) with lupus nephritis, or CTNTI (7) with demyelinating polyneuropathy and myositis. The authors also discussed in detail medications, infectious triggers and malignancies and finally suggested the implementation of an antigen-based classification in place of the simplified distinction between primary and secondary MN. For practical use, an interesting algorithm has been proposed that distinguishes between anti-PLA2R positive and negative forms and, in the latter case, considers the age of presentation (SEMA3B is frequent in pediatric cases), the association with other autoimmune conditions (EXT1/2, NCAM1, TGFBR3 with lupus and CTNTI with neuropathy/myositis) and, finally, the IgG pattern (segmental IgG suggests NELL1-, global IgG indicates THSD7A-, PCDH7- or HTRA1-associated MN).

Furthermore, De Menezes Neves et al. and He et al. described two particular forms of MN associated with Grave’s disease and IgA nephropathy, respectively, which are rare occurrences but seem to represent an overlap syndrome between two distinct immune disorders. MN associated to Graves’ disease was histologically characterized by thyroglobulin deposition along the capillary loop. Interestingly, proteinuria normalized only after radioiodine therapy (De Menezes Neves et al.), indicating potential necessity of a thyroid assessment in patients with MN. The huge population with IgA/MN (137 patients) had a lower median level of galactose-deficient IgA1 and were less proteinuric than MN(He et al.).

Despite these advances and the comprehensive clinical characterization of the role of antibodies for diagnosing and monitoring patients with MN, insights on pathogenic molecular mechanisms are still limited. Current research focuses on the characterization of the B cell response with the identification of new antigenic targets as well as the role of the complement system. In addition, there are many arguments in favor of an involvement of T cellular immunity in the pathogenesis of MN. Zhao et al. reviewed the role of follicular helper T cells in the pathogenesis of MN and summarized many studies showing the orientation towards the Th17 pathway in MN, paving the way for new targeted therapies.

## Minimal change disease and FSGS

The molecular mechanisms underlying MCD and primary FSGS are only partially defined. Both entities are considered diseases of podocytes, or podocytopathies, that are characterized by podocyte foot process effacement, loss of podocyte architecture and slit diaphragm integrity in the absence of any inflammatory hallmarks. Both conditions are considered of immunologic origin and linked to T and B cells, and more recently also to autoimmune processes. Secondary forms also exist as part of more complex settings, such as viral infections (e.g. cytomegalovirus, Epstein Barr virus, SARS-CoV2) and cancer, notably Hodgkin’s lymphoma, may be associated with MCD (14).

The theory on T cell involvement dates back to 1974 (Shalloub hypothesis) and was based on the favorable response to steroids and on the association of MCD with T cell malignancies (15). However, T cell involvement has not been confirmed across studies (16–18). Experimental induction of proteinuria by injection of supernatant from a T cell hybridoma into rats was a part of the Shalloub hypothesis (19) and served as the basis to the hypothesis on the existence of a still uncharacterized “extra renal” circulating glomerular permeability factor (20). An implication of Treg is indirectly suggested by the association of MCD/primary FSGS (but also of MN) with IPEX, a FoxP3 X-linked congenital immune pathology characterized by altered Tregs, polyendocrinopathy and enteropathy (21, 22). Treg expansion with drugs, e.g. IL2 and anti-CD20, is not associated with a clear anti-proteinuric effect in patients with MCD/FSGS (23–25) limiting the idea on a direct Treg involvement. More recently, a growing body of evidence linked MCD/primary FSGS pathogenesis to B cells and also autoimmunity. An involvement of B cells is supported by the increasing and succesful use of anti-CD20 monoclonal antibodies in both conditions (26). Autoimmunity is linked to the observation of several antibodies in the serum of patients with MCD/primary FSGS, such as anti-nephrin, anti-annexin A2 and anti-UCHL1 antibodies (11, 27, 28).

Two papers in this issue addressed the crucial aspect of T/B cells subsets in MCD. Fribourg et al. utilized time of flight mass cytometry (CyTOF) for studying patients with steroid-dependent nephrotic syndrome treated with either chimeric or human anti-CD20 monoclonal antibodies. CyTOF utilizes metal isotopes as cell surface markers that display specific mass spectrometry signatures for simultaneous quantification of over 40 circulating cells. The authors confirmed previous findings on a significantly higher number of switched memory B cells in relapsing patients (29) and identified among 5 subsets of switched memory B cells, IgD- CD27+ CD38+ CD95+ antibody-secreting cells as the subset most strongly associated with disease relapse. Positivity for CD38 of this cell subset is of particular interest since it is a marker of plasma cells and immature plasmablasts (30) and is absent in memory B cells. A second interesting finding of the study above was that patients undergoing disease relapse had a faster recovery of B_REG_ that are cells capable of inhibiting the T cell compartment through IL10 and IL35. Major modifications of T cells were not found.

The exact molecular mechanism underlying the B cell-mediated detrimental effect in children and adult patients with MCD/primary FSGS remain to be determined and the accurate mechanisms associated with therapeutic efficacy of B cell-depleting agents are not well elucidated. In a second paper, Colucci et al. discussed current knowledge regarding the role of B cell dysregulation in the pathogenesis of MCD/FSGS in both pediatric and adult patients. This review summarized the most relevant clinical and experimental findings suggesting a key role of B lymphocytes in the pathogenesis of MCD/primary FSGS. Strikingly, at onset disease, during the first episode of nephrotic syndrome, alteration in B cell homeostasis and distribution of B cell subpopulation seem to be widely different in children compared to adult populations. The production of pathogenic autoantibodies (anti-nephrin and anti-UCHL1) targeting podocytes and/or slit diaphragm structure but also the secretion of B cell-derived cytokines may play a crucial role in the increase of glomerular capillary permeability leading to podocyte cytoskeleton disorganization and proteinuria. In addition, the B cell production of hyposialylated IgM directed against T cells may effect on corticosteroid response.

## Therapies: Anti-CD20 monoclonal antibodies and more

Scolari et al. reviewed current treatment strategies for MN. They presented data on the comparison between drugs utilized in early 2000 that is historically known as the Ponticelli regimen (cyclophosphamide plus steroids) with more recent approaches that utilize anti-CD20 monoclonal antibodies (e.g. rituximab). The conclusion was that the two treatments had comparable effects opening *de facto* the discussion on which therapies should be utilized first.

In addition, original work by Teisseyre et al. demonstrated the importance of rituximab plasma levels 3 months after rituximab infusion for remission induction in MN. They also highlighted other mechanisms that may limit rituximab efficacy (e.g. anti-rituximab antibodies). Teisseyre et al. additionally reviewed in this issue the recent therapeutic advances in MN, the mechanisms for rituximab resistance, and proposed personalized management based on immunomonitoring of rituximab, anti-rituximab antibodies and autoantibodies.

The knowledge on autoantibodies targeting specific renal proteins in patients with MN principally enables the use of antigen-specific treatments. Such treatments would ideally target the immunological disease mechanisms while sparing protective immunity, thus bearing an enormous potential to enhance specificity, efficacy and compatibility. The gap between the increasing knowledge on the pathogenic role of autoantibodies and autoantigens in MN on the one side and the currently available treatments with limited specificity on the other side was discussed in the perspective by Köllner et al.. The authors highlight two potential strategies targeting both the pathogenic antigens and the antibody-producing B cells.

The use of chimeric anti-CD20 in MCD/FSGS has been established over the last decade with good results. Basu et al. extended to the use of the human anti-CD20 antibody ofatumumab that was developed in substitution of chimeric products with the hope to improve therapeutic efficiency and reduce risks. A first report indicated that ofatumumab utilized in very high doses (about 10 times higher than rituximab) successfully normalized proteinuria in 6 patients with FSGS resistant to other drugs (31). This finding has never been confirmed in other studies, probably for the sake of side effects linked with the very high dosage. When ofatumumab was utilized in doses that doubled rituximab, no effects in patients with multi-drug resistant nephrotic syndrome were achieved (32). A recent randomized clinical study comparing ofatumumab and rituximab in patients with steroid and/or multidrug dependence showed no superiority of the former in comparison with rituximab and a better outcome of children under 9 years treated with the chimeric molecule (25).

## Conclusions

In conclusion, this special issue on ‘Immune Dysfunction in Nephrotic Syndrome’ contains important and timely manuscripts that covered years of research in the broad area of nephrotic syndrome. New antibodies involved in MN, therapy evolutions with anti-CD20 monoclonal antibodies and new proposals for biomarkers and targeted treatments have been major topics of interest. Proposals for new drugs have also been presented and mechanisms of resistance to drugs that are currently utilized have been discussed. Overall, this issue witnesses the evolution made in recent years and opens to new discoveries that will complete the knowledge on molecular mechanisms in different conditions associated with nephrotic syndrome and, we hope, consolidate new therapeutic approaches.

## Author contributions

All authors listed have made a substantial, direct, and intellectual contribution to the work and approved it for publication.

## Conflict of Interest

VA received consulting fees from Addmedica, Sanofi Genzyme, Travere, Alnylam, and Astrazeneca outside of the submitted work.

The remaining authors declare that the research was conducted in the absence of any commercial or financial relationships that could be construed as a potential conflict of interest.

## Publisher’s note

All claims expressed in this article are solely those of the authors and do not necessarily represent those of their affiliated organizations, or those of the publisher, the editors and the reviewers. Any product that may be evaluated in this article, or claim that may be made by its manufacturer, is not guaranteed or endorsed by the publisher.
